# Immunohistochemical Expression of Differentiated Embryonic Chondrocyte 1 and Cluster of Differentiation 44 in Oral Potentially Malignant Disorders

**DOI:** 10.3390/medicina61020251

**Published:** 2025-02-01

**Authors:** Bianca-Andreea Onofrei, Delia Gabriela Ciobanu Apostol, Mădălina-Gabriela Tanasă, Elena-Raluca Baciu, Cristina Popa, Ana Maria Sciuca, Mihaela Paula Toader, Victor-Vlad Costan

**Affiliations:** 1 Department of Surgicals, Faculty of Dental Medicine, “Grigore T. Popa” University of Medicine and Pharmacy, 700115 Iasi, Romania; bianca.onofrei@umfiasi.ro (B.-A.O.); cristina.popa@umfiasi.ro (C.P.); ana.filioreanu@umfiasi.ro (A.M.S.); mihaela.toader@umfiasi.ro (M.P.T.); victor.costan@umfiasi.ro (V.-V.C.); 2 Department of Morpho-Functional Sciences I, Faculty of Medicine, “Grigore T. Popa” University of Medicine and Pharmacy, 700115 Iasi, Romania; tanasa.madalina-gabriela@email.umfiasi.ro; 3 Department of Implantology, Removable Prostheses, Dental Technology, Faculty of Dental Medicine, “Grigore T. Popa” University of Medicine and Pharmacy, 700115 Iasi, Romania; elena.baciu@umfiasi.ro

**Keywords:** oral leukoplakia, oral lichen planus, actinic cheilitis, oral squamous cell carcinoma, DEC1, CD44

## Abstract

*Background and Objectives*: Oral cancer remains a critical global health concern, with oral squamous cell carcinoma (OSCC) being the most prevalent form. Oral potentially malignant disorders (OPMDs), such as oral leukoplakia (OLK), oral lichen planus (OLP), and actinic cheilitis (AC), often precede OSCC. Identifying reliable biomarkers is vital for assessing malignant transformation risk. The present study aimed to evaluate the immunohistochemical expression of differentiated embryonic chondrocyte 1 (DEC1), a marker of dysplasia severity, and cluster of differentiation 44 (CD44), which is associated with cancer progression, in OPMD and OSCC tissues. *Materials and Methods*: A retrospective analysis was conducted on 145 biopsy specimens from January 2015 to January 2023, comprising normal mucosa (NM), OLK, OLP, AC, and OSCC. DEC1 and CD44 expression levels were assessed using immunohistochemical staining. Positivity scores were determined based on staining intensity and extent, with statistical analyses performed using SPSS software (SPSS Inc., Chicago, IL, USA, version 29.0 for Windows). *Results*: It was found that CD44 expression significantly increased across OPMD and OSCC compared to NM (*p* < 0.001). Conversely, DEC1 expression was consistent across lesion types and dysplasia levels. CD44 expression was the highest in AC and OSCC, underscoring its potential role as a progression marker. *Conclusions*: The results indicate that CD44 is a more sensitive marker for assessing dysplastic severity and malignant transformation, while DEC1 may serve as a complementary marker for early-stage evaluation. Further research involving larger cohorts is needed to confirm these findings.

## 1. Introduction

Oral cancer continues to pose a significant global public health challenge despite progress in oncology. Each year, over 300,000 new cases are reported, with oral squamous cell carcinoma (OSCC) being the most common form of oral cancer, characterized by high morbidity and mortality rates, with a 5-year survival rate below 50% [1,2]. In 2005, the WHO replaced the terms “precancerous conditions” and “precancerous lesions” with “oral potentially malignant disorders” (OPMDs), which describe lesions or conditions with a risk of malignancy either at diagnosis or in the future [3]. Most of these oral carcinomas develop from oral lesions with malignant transformation potential.

Lesions such as oral leukoplakia (OLK), oral erythroplakia, oral lichen planus (OLP), oral submucous fibrosis, and actinic cheilitis (AC) are widely recognized for their malignant transformation potential [4,5,6,7]. Leukoplakia, defined as a hyperkeratotic (white) plaque/patch of mucosa with monoclonal proliferation, is associated with dysplastic changes in 40% of cases and is classified as keratinizing dysplasia, with an annual transformation risk of 1.36–2.9% [5]. Oral lichen planus is a chronic inflammatory mucocutaneous lesion mediated by T lymphocytes, with uncertain etiology and pathogenesis. It is characterized by recurrences and remissions and is often located on the buccal mucosa with multifocal, bilateral, and symmetrical involvement. Clinically, it presents as white, lace-like lesions (Wickham’s striae), sometimes accompanied by atrophic or erosive areas [8]. Actinic cheilitis is a common premalignant lesion located on the vermilion of the lower lip, resulting from chronic ultraviolet light exposure. Clinically, it manifests as blurring of the vermilion border, erythema, edema, dry scaling, and persistent crusting, sometimes accompanied by leukoplakia [9,10].

Recent predictive markers for evaluating the malignant transformation risk of oral precancerous lesions include differentiated embryonic chondrocyte 1 (DEC1), cluster of differentiation 44 (CD44), aldehyde dehydrogenase 1 (ALDH1), Maspin, and E-cadherin.

DEC1, a transcription factor from the basic helix–loop–helix (bHLH) family, controls various cellular processes like the circadian rhythm and cell cycle progression, and it has a complex role in cancer. It can act as either a tumor suppressor, like in lung cancer, where it suppresses tumor growth by inducing G1 arrest via cyclin-dependent kinase inhibitor (CDKI) p21, or as an oncogene, like in breast cancer, promoting cell survival and apoptosis resistance [11]. DEC1 is also associated with hypoxia, upregulating vascular endothelial growth factor (VEGF) for angiogenesis and tumor growth, particularly through interactions with hypoxia-inducible factor-1α (HIF-1α), and it promotes epithelial–mesenchymal transition (EMT) in gastric cancer, enhancing metastatic potential by upregulating transcription factors like Snail and Twist [12].

CD44 is a cell surface glycoprotein, which is involved in cell–cell and cell–matrix interactions, with a key role in tumor biology, particularly in cancer stem cells (CSCs), influencing their self-renewal, differentiation, tumor initiation, metastasis, and therapy resistance [13]. There are two isoforms for CD44, standard (CD44s) and variant (CD44v), with CD44v being involved in metastasis, epithelial-to-mesenchymal transition (EMT), and the adaptive plasticity of cancer cells [14]. By interacting with hyaluronan, CD44 activates signaling pathways (PI3K/Akt, Ras/MAPK), promoting CSC survival, and aids in immune evasion, suppressing immune responses and promoting immune escape via transforming growth factor beta (TGF-β) and PD-1/PD-L1 pathways [15].

Both CD44 and DEC1 are pivotal in tumor biology, each contributing through distinct but often overlapping mechanisms, and together, they influence cancer stemness, metastasis, and resistance to therapy, highlighting their potential as therapeutic targets in cancer treatment. Recent research emphasizes the need to further explore their interactions and downstream pathways to develop more effective strategies for cancer management [16].

This study aimed to evaluate the immunohistochemical expression levels of DEC1 and CD44 in tissue specimens obtained from patients diagnosed with three of the most common oral premalignant lesions (oral leukoplakia, oral lichen planus, and actinic cheilitis), as well as with oral squamous cell carcinoma. To address this objective, we formulated the following null hypotheses: there are no significant differences in the DEC1 and CD44 immunoscores among patients with OLK, OLP, and AC; no significant differences in the DEC1 and CD44 immunoscores across the various dysplastic levels of oral lesions; and no significant differences in the DEC1 and CD44 immunoscores between normal mucosa, OPMDs, and OSCC.

## 2. Materials and Methods

### 2.1. Research Design

Biopsy tissue specimens obtained between January 2015 and January 2023 were retrieved from the archives of the ”Sf. Spiridon” Emergency Clinical Hospital in Iași, Romania, Pathology Department. This study was approved by the Ethics Committees of the “Sfântul Spiridon” Emergency Clinical Hospital in Iași, Romania (No. 35/24.04.2023) and the “Grigore T. Popa” University of Medicine and Pharmacy in Iași (No. 320/5.06.2023).

A total of 145 formalin-fixed, paraffin-embedded tissue blocks were selected for the study (Table 1). For the control group (normal mucosa specimens), non-inflamed tissue collected during biopsy was utilized. The diagnoses of oral leukoplakia, oral lichen planus, actinic cheilitis, and OSCC were confirmed by the Pathology Department. Additionally, the lesions were reevaluated by two oral pathologists to ensure diagnostic accuracy. All patients provided informed consent for the use of their tissues in research.

The oral lesions were classified based on the location and types of dysplasia (0—no dysplasia; 1—mild dysplasia; 2—moderate dysplasia; 3—severe dysplasia) [17].

### 2.2. Immunohistochemical Analysis

Formalin-fixed, paraffin-embedded tissue blocks were sectioned into 4 μm slices for immunohistochemistry (IHC) staining of CD44 and DEC1. The mouse- and rabbit-specific horseradish peroxidase/3,3′-diaminobenzidine (HRP/DAB) detection IHC Kit (Abcam, Cambridge, UK) was used according to the manufacturer’s protocol. Briefly, tissue sections were deparaffinized in three successive xylene baths (1.5 min each) and rehydrated in three successive ethanol baths (1.5 min each), followed by heat-induced antigen retrieval in a microwave at 97–99 °C for 30 min.

Endogenous peroxidase activity and nonspecific background staining were blocked by incubating the slides in Peroxid Block (10 min) and Protein Block (10 min) solutions, respectively. The sections were then incubated overnight at 4–6 °C, with either a rabbit polyclonal antibody against human DEC1 (BHLHE40) (1:100, Antibodies Online, Aachen, Germany) or a mouse monoclonal antibody against CD44 (1:500, C44Mab-5 clone, Abcam). Afterward, the sections were incubated with Biotinylated Goat Anti-Polyvalent (10 min) and Streptavidin Peroxidase (10 min) solutions, respectively. Development (staining) was performed using DAB (30 μL DAB Chromogen in 1.5 mL DAB substrate, 1–2 min). In this method, the biotinylated secondary antibody binds to the primary antibody, and HRP-labeled streptavidin binds to the secondary antibody. This interaction produces a brown-colored precipitate at the site of primary antibody binding upon reaction with DAB.

Finally, the slides were counterstained with hematoxylin (1 min), dehydrated in three consecutive ethanol baths (1.5 min each), clarified with xylene (3 min), coverslipped, and observed under a microscope [18].

### 2.3. Antibodies

The commercial antibodies that were used are presented in Table 2.

**Figure 1:**
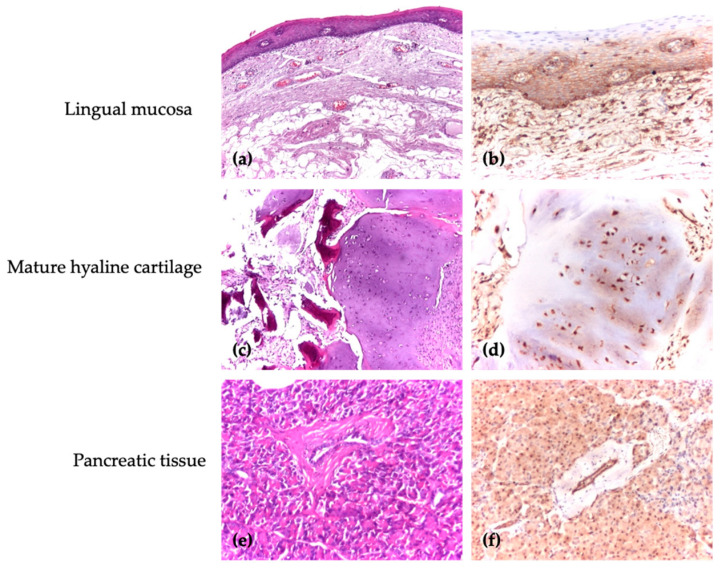
The positive control of reaction for DEC1 and CD44. (**a**) Control tissue, HE, ×40; (**b**) control tissue, uniform membrane expression (Ab. anti-CD44), ×40; (**c**) control tissue, HE, ×40; (**d**) control tissue, chondrocyte nuclear expression (Ab. anti-DEC1), ×100; (**e**) control tissue, HE, ×40; (**f**) control tissue, nuclear expression of acini and ducts (Ab. anti-DEC1), ×100.

For each antibody, positive and negative control markers for the immunohistochemical reaction were used. In accordance with the control tissues specified in the data sheet, the positive control for CD44 was the lingual mucosa, where a specific membrane marker was identified, and for DEC1, the positive control was in cartilaginous and pancreatic tissue, where the antibody showed a specific nuclear marker. The negative control consisted of a separate slide with the patient’s test sample, to which no primary antibody was applied (Figure 1).

### 2.4. Evaluation of Score

The immunohistochemical stains were independently evaluated by two pathologists by selecting 10 high-power representative fields and assessing the expression of DEC1 and CD44 proteins. The analysis considered both the extent of staining (percentage of positive cells) and its intensity. Expression of DEC1 and CD44 was considered positive when the scores were ≥3 [19] (Table 3). A semiquantitative analysis of antibody expression was performed in epithelial cells under 40× magnification using the following criteria: in ten high-power fields, the proportion of positive cells was recorded and calculated as (number of positive cells in a field/total number of cells) × 100 [20]. Two pathologists independently scored the slides, compared their results, and discussed any discrepancies. This consensus approach was valuable in reaching a final score and minimizing bias. In all cases, the interpretation of immunohistochemical reactions was performed in comparison with areas of normal morphology, which served as controls for each patient.

### 2.5. Statistical Analysis

Descriptive statistical analysis was performed using IBM Statistical Package for the Social Sciences (SPSS) software (SPSS Inc., Chicago, IL, USA, version 29.0 for Windows). Differences between groups were assessed using the Chi-squared and Kruskal–Wallis tests, with the statistical significance threshold set at 5% (*p* < 0.05).

## 3. Results

### 3.1. The Characteristics of the Study Specimens

**Figure 2:**
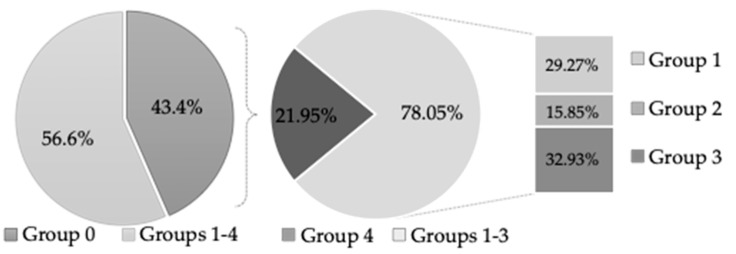
The characteristics of the study specimens.

Of the retrieved specimens, 82 (56.6%) were taken from areas with malignant or oral potentially malignant disorders, while the remaining 63 (43.4%) were from normal mucosa (Figure 2).

Among the 18 tissue specimens diagnosed with OSCC, 72.2% were classified as well-differentiated forms, while the remaining 27.8% were categorized as moderately differentiated.

A higher frequency of OLK was observed in the lingual mucosa (33%), OLP in the buccal mucosa (84.6%), and OSCC in the lower labial mucosa (61.1%), while all AC cases were located in the lower labial mucosa (100%). Statistically significant differences were observed among the four studied groups based on location (Table 4).

### 3.2. Comparison of DEC1 and CD44 Immunoscores Among Patients with Oral Leukoplakia, Oral Lichen Planus, and Actinic Cheilitis

The DEC1 extent of positivity scores was distributed relatively evenly across the three lesion groups, with 46.2% of OLP cases showing a score of 0, while 55.6% of AC cases had scores of 3 or 4. In OLK, the scores were more evenly spread. No OLP specimens displayed a DEC1 intensity of positivity score of 3, which was observed in only one specimen each, from OLK and AC. However, DEC1 expression did not show statistically significant differences between the lesion types. In contrast, CD44 scores varied significantly across the groups. A majority of OLK (75%) and AC (96.3%) specimens had CD44 scores of 2 or 3, while most OLP specimens had scores of 0 or 1. CD44 expression was the highest in AC (mean 6.22 ± 2.154; median 6), followed by OLK (mean 4.87 ± 2.894; median 6), with OLP showing the lowest values (mean 2.38 ± 2.293; median 1). These findings highlight significant differences in CD44 expression across lesion types, unlike DEC1, which showed no significant variation (Table 5).

### 3.3. Comparison of DEC1 and CD44 Immunoscores Across Different Types of Oral Lesions and Levels of Dysplasia

The analysis revealed that the CD44 immunoscore was lower in OPMDs without dysplasia, and it was almost double in patients with types 1, 2, or 3. In contrast, the lowest values of the DEC1 immunoscore were observed in patients with severe dysplasia, while the highest values were observed in patients with mild dysplasia (Table 6).

**Figure 3:**
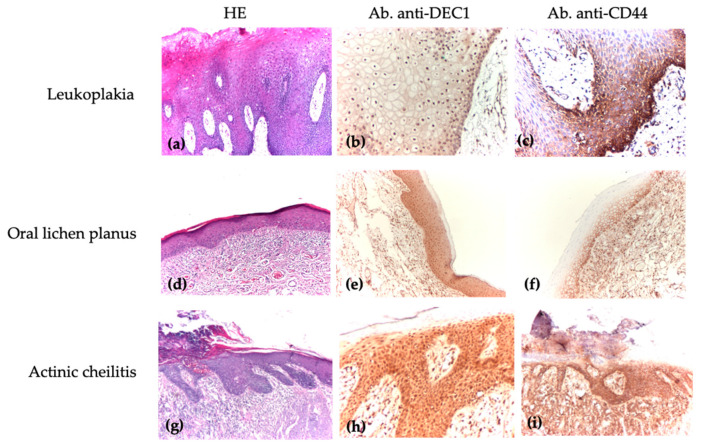
Immunohistochemical analysis of OPMD. (**a**) Leukoplakia with low dysplasia, affecting one-thirds of thickness of epithelium (lower part of epithelium, center, and down), associated with lympho-plasmacytic inflammatory infiltrate in band, HE, ×40. (**b**) Leukoplakia—nuclear expression, in significant number of cells, high intensity (Ab. anti-DEC1), ×100. (**c**) Leukoplakia—membrane expression with strong staining intensity in one-thirds of lower part of mucosa in 20% of cells (Ab. anti-CD44), ×100. (**d**) Oral lichen planus without dysplasia, HE, ×40. (**e**) Oral lichen planus–nuclear expression with moderate staining intensity in 90% of cells (Ab. anti-DEC1), ×40. (**f**) Oral lichen planus—membrane expression with high intensity in 40% of cells in lower part of mucosa (Ab. anti-CD44), ×40. (**g**) Actinic cheilitis with moderate dysplasia, HE, ×40. (**h**) Actinic cheilitis—increased cell count, high nuclear staining intensity and diffuse in all mucosa (Ab. anti-DEC1), ×100. (**i**) Actinic cheilitis—high, diffuse membranous staining in all layers of mucosa (Ab. anti-CD44), ×40.

Oral potentially malignant disorders, directly associated with the malignant tumor process, were identified in our study. These lesions were either coexistent with malignant lesions or located in close proximity to the tumor. A histopathological examination revealed varying degrees of cytological and architectural dysplasia proportional to the severity of the dysplasia (Figure 3a,d,g).

In oral leukoplakia, the IHC reaction with anti-DEC1 (Figure 3b) showed a cellular distribution of 25–74% in most cells with weak to moderate intensity, while anti-CD44 (Figure 3c) demonstrated strong staining intensity within the same range of distribution.

For oral lichenoid lesions, the IHC reaction with anti-DEC1 (Figure 3e) revealed a distribution of less than 5% in most cells, with weak to moderate intensity (46.2% with no staining and 53.9% with weak to moderate staining). Anti-CD44 (Figure 3f) also showed weak intensity with a similar cellular distribution.

In actinic cheilitis, the IHC reaction with anti-DEC1 (Figure 3h) and anti-CD44 (Figure 3i) indicated a cellular distribution of 50–74% in most cells, with weak and strong staining intensity, respectively.

### 3.4. Analysis of DEC1 and CD44 Immunoscores Between Normal Mucosa, Oral Premalignant Lesions, and Oral Squamous Cell Carcinoma

The median and mean values of DEC1 and CD44 immunoscores in NM, OPMD, and OSCC are presented in Table 7 and Table 8.

**Figure 4:**
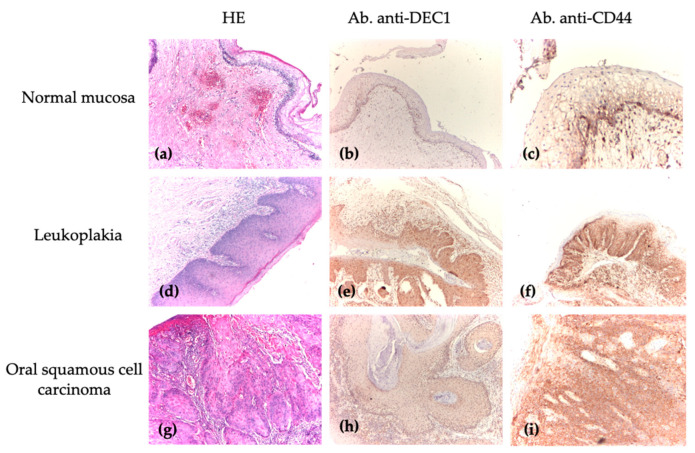
Immunohistochemical analysis of NM, OLK, and OSCC. (**a**) Normal mucosa, HE, ×40; (**b**) normal mucosa—absent expression (Ab. anti-DEC1), ×40; (**c**) normal mucosa—low cell count, weak staining intensity, in lower part of mucosa, in basal layer (Ab. anti-CD44), ×40; (**d**) leukoplakia without dysplasia, HE, ×40; (**e**) leukoplakia—nuclear expression, in 40% of cells, moderate intensity, upper right (Ab. anti-DEC1), ×40; (**f**) leukoplakia—membrane expression with strong staining intensity in 60% of cells (Ab. anti-CD44), ×40; (**g**) moderate differentiated, ulcerated, infiltrative OSCC with keratin pearls, HE, ×40; (**h**) well-differentiated OSCC—nuclear expression, diffuse, most cells, weak intensity (Ab. anti-DEC1), ×40; (**i**) well-differentiated OSCC—membrane expression, increased cell number, strong intensity (Ab. anti-CD44), ×40.

A morphological analysis using routine HE staining of normal mucosa, compared to premalignant and malignant oral lesions, revealed specific cytoarchitectural and architectural differences characteristic of the normal histological structure of oral mucosa versus certain lesion entities (Figure 4a,d,g). The IHC reaction with anti-DEC1 (Figure 4b) and anti-CD44 (Figure 4c) antibodies showed a distribution between 0 and 24% in most cells, with weak intensity or an absence of a reaction in biopsy specimens with normal mucosa. In comparison to premalignant and malignant oral lesions, the cellular distribution predominantly ranged between 25 and 74% with weak to moderate intensity (Figure 4e,h) and between 50 and 100% with strong intensity, respectively (Figure 4f,i).

## 4. Discussion

The results of this study show that the first null hypothesis could be accepted for DEC1 but had to be rejected for CD44. While the second null hypothesis was accepted, the findings reveal a noticeable difference in the behaviors of DEC1 and CD44 across various types of dysplasia. DEC1 demonstrated limited utility in distinguishing between stages. In contrast, CD44 immunoscores increased in type 3 dysplasia specimens, registering values almost twice as high as those observed in type 0 dysplasia. This finding in CD44 suggests that it is a more sensitive marker for dysplasia severity and malignant progression. Similar results were obtained by Mirhashemi et al. [21], who investigated CD44 and CD24 expression in OSCC and oral epithelial dysplasia. High levels of these markers were observed in both conditions, emphasizing the need for a detailed examination of dysplastic lesions to predict malignant transformation [21]. Subsequently, Thankappan et al. [19] analyzed the expression of CD44, ALDH1, OCT4, and SOX2 in various grades of oral epithelial dysplasia. The study included 35 samples of oral epithelial dysplasia (from patients diagnosed with leukoplakia, erythroplakia, and oral submucous fibrosis), divided into mild, moderate, and severe grades, as well as 10 samples of normal epithelium. The results show a progressive and significant increase in CD44 expression as dysplasia severity increased (*p* < 0.05). This suggests that CD44 plays a critical role in malignant progression and could be used to identify patients at high risk of malignant transformation. The study highlighted the importance of using CD44 as a predictive marker for assessing risk and monitoring oral epithelial dysplasia. A study conducted by Zargaran et al. [22] investigated the expression levels of beta-catenin (β-catenin) and CD44 in 55 samples, including cases of epithelial hyperplasia, OLP, and OSCC. Their analyses, using both quantitative and semi-quantitative methods, identified significant variations in marker expression among the three types of lesions. The membranous expression of CD44 and the nuclear/cytoplasmic levels of β-catenin were found to be lower in OSCC compared to OLP, indicating that these markers can help distinguish the behavior of OLP and OSCC. Similarly, Abdal et al. [23] assessed the premalignant potential of oral leukoplakia (OLK), OLP lesions, and OSCC through the expression of CD44 and E-cadherin. CD44 was observed in 40% of OLK and 50% of OLP samples, while only 30% of OSCC samples expressed this marker, though the staining intensity of CD44 and E-cadherin was not significantly different (*p* < 0.16). However, 70% of OSCC cases exhibited mild to moderate expression intensity, which was statistically significant compared to OLK and OLP (*p* < 0.004). These findings suggest that changes in CD44 and E-cadherin expression can be indicative of dysplasia and the premalignant nature of lesions such as OLK and OLP when compared to oral carcinomas.

The results of this study highlight significant differences in the characteristics of DEC1 and CD44 markers between normal epithelium, oral potentially malignant lesions, and malignant lesions, leading to the rejection of the third null hypothesis. These findings indicate that both markers play a significant role in tumor progression, with their immunoscores progressively increasing from normal epithelium to malignant lesions. CD44 showed a more pronounced variation, suggesting its higher relevance in assessing tumor aggressiveness.

Mao et al. [24] investigated DEC1 expression in normal oral mucosa, oral leukoplakia, and oral squamous carcinoma, aiming to assess its role in progression from healthy to malignant tissues. DEC1 was minimally expressed in normal epithelium but significantly increased in OLK, particularly in moderate and severe dysplasia (*p* < 0.05). Its highest expression was observed in OSCC, correlating with tumor aggressiveness and invasive potential. The study concluded that DEC1 is a valuable biomarker for identifying high-risk precancerous lesions and assessing malignant progression. Based on the analysis of data from the literature, we identified a limited number of studies focused on both markers, DEC1 and CD44, and noted potential discrepancies in the reported results, which appear to vary depending on the specific context analyzed.

Clinical studies have shown a significant correlation between high CD44 levels and poor prognosis. This marker is linked to a higher rate of regional metastases, tumor aggressiveness, and reduced survival rates in patients undergoing radiotherapy, suggesting its role in therapy resistance and unfavorable outcomes [25,26]. Saghravanian et al. [27] correlated the immunoexpression levels of CD44 and p63 markers with clinical parameters in OSCC. Both markers were expressed at higher levels in advanced and high-grade OSCC cases. CD44 was noted for its potential role in tumor initiation and progression, aligning with cancer stem cell theories. However, no correlation was found with patient demographics, such as age or gender. Similarly, Singh et al. [28] demonstrated, using qRT-PCR and ELISA techniques, elevated mRNA and CD44 levels in samples collected from patients with OSCC. These results highlight a correlation between CD44 immunoexpression, increased tumor burden, and metastatic potential, further solidifying the role of CD44 as a marker of tumor progression.

In contrast, the study conducted by Dhumal et al. [29] analyzed, in detail, the expression of CD44 and ALDH1, focusing on their role in the malignant transformation of OPMD and their involvement in lymph node metastases in oral squamous cell carcinoma (OSCC). The results show significant differences in the expression of these markers between normal epithelium, OPMD, and OSCC, offering new perspectives on tumor progression. The percentage of CD44-positive cells varied significantly depending on the degree of tumor differentiation. The study revealed that the average number of CD44-immunopositive cells was greater in normal mucosa compared to OPMD without dysplasia, as well as low-risk dysplasia, high-risk dysplasia, and OSCC. Moreover, severe dysplasia and OSCC cases exhibited decreased CD44 expression. The relationship between dysplasia grade and reduced CD44 expression was linked to key biological processes, including proliferation, cell differentiation, motility, and tumor invasion. Besides CD44, the study also analyzed ALDH1 expression, which proved to be an important marker for the malignant transformation of OPMD, especially in high-risk dysplasia cases. The results suggest that CD44 is useful for assessing lymph node metastases and stratifying the degree of tumor differentiation, while ALDH1 may serve as a strong indicator of malignant transformation risk in OPMD.

Incorporating CD44 scoring into clinical workflows, as an indicator of the malignant transformation of OPMDs, could significantly improve diagnostic accuracy and treatment planning. It enables the identification of high-risk patients and supports the implementation of targeted therapies or monitoring protocols. Furthermore, combining DEC1 with other biomarkers (e.g., ALDH1, SOX2, Maspin, β-catenin, or E-cadherin) may enhance specificity and sensitivity, enabling a multifaceted approach to patient assessment and advancing personalized therapeutic strategies.

This study had several noteworthy limitations. First, the use of distinct patient groups rather than longitudinal data from the same individuals posed challenges in establishing causality, as this approach does not account for interindividual biological variability that may influence the observed outcomes. Additionally, the role of oral microbiota, which is increasingly recognized for its critical contribution to immune regulation and oral carcinogenesis, was not directly investigated, representing an area for future exploration.

Furthermore, the retrospective nature of this study, based on data collected from a single center, limits the generalizability of the findings to broader populations. To address these limitations, future research should consider multicenter studies with a prospective design, integrating longitudinal data and exploring the impact of oral microbiota, to provide a more comprehensive and widely applicable understanding of the observed phenomena.

## 5. Conclusions

The findings of this study provide valuable insights into the roles of DEC1 and CD44 in the diagnosis and management of premalignant and malignant oral lesions, as outlined below:

- The analysis of DEC1 and CD44 markers revealed important differences, demonstrating their complementary roles in diagnosing and managing premalignant and malignant oral lesions;
- CD44 expression progressively increased from normal epithelium to dysplastic lesions and carcinoma, highlighting its relevance for the early diagnosis of dysplasia;
- Analyzing DEC1, as a marker of early-stage lesions, and CD44, as an indicator of tumor progression, is a possible synergistic approach for optimal clinical management.

However, further studies with larger cohorts, methodological standardization, and longitudinal evaluations are needed to validate and expand the applicability of these markers. Considering the limited data available in the recent literature, our findings contribute to a better understanding of the molecular roles of DEC1 and CD44 in oral carcinogenesis, offering new perspectives for diagnosis, prognosis, and monitoring.

## Figures and Tables

**Table 1:** Study groups.

Group	Diagnosis	Number
Group 0—control group	Normal mucosa (NM)	63
Group 1	Oral leukoplakia (OLK)	24
Group 2	Oral lichen planus (OLP)	13
Group 3	Actinic cheilitis (AC)	27
Group 4	Oral squamous cell carcinoma (OSCC)	18
		Total = 145

**Table 2:** Antibodies used in the evaluation of premalignant and malignant lesions of the oral cavity.

Antibody	Clone	pH	Class	Dilution	Expression
DEC1 (Antibodies Online, Aachen, Germany)	BHLHE40	9	rabbit, polyclonal	1:100	nuclear
CD44 (ABCAM, Cambridge, UK)	C44Mab-5	9	mouse, monoclonal	1:500	membrane

**Table 3:** Semi-quantitative scores for DEC1 and CD44 [18,19].

Immunohistochemical Staining	Score	
Extent of Positivity	1	5–24%
	2	25–49%
	3	50–74%
	4	75–100%
Intensity of Positivity	0	no staining
	1	weak staining
	2	moderate staining
	3	strong staining
Final Immunoscores = Extent of Positivity × Intensity of Positivity (resulting in a range from 0 to 12)		

**Table 4:** Lesion locations between study groups.

Location	Histopathological Diagnosis								Pearson Chi-Squared Test
	Group 1		Group 2		Group 3		Group 4		
	N	%	N	%	N	%	N	%	
Lower labial mucosa	7	29.2%	-	-	27	100.0%	11	61.1%	Chi2 = 79.826 *p* < 0.001 *
Buccal mucosa	3	12.5%	11	84.6%	-	-	-	-	
Lingual mucosa	8	33.3%	1	7.7%	-	-	4	22.2%	
Other locations	6	25.0%	1	7.7%	-	-	3	16.7%	

* Statistical significance, *p* < 0.05.

**Table 5:** Intergroup comparison of extent of positivity, intensity of positivity, and immunoreactive scores.

			Histopathological Diagnosis						Pearson Chi-Squared Test
			Group 1		Group 2		Group 3		
			N	%	N	%	N	%	
Extent of positivity	DEC1	0	5	20.8%	6	46.2%	2	7.4%	Chi2 = 13.725 *p* = 0.089
		1	4	16.7%	1	7.7%	3	11.1%	
		2	7	29.2%	3	23.1%	7	25.9%	
		3	5	20.8%	2	15.4%	14	51.9%	
		4	3	12.5%	1	7.7%	1	3.7%	
	CD44	0	1	4.2%	2	15.4%	-	-	Chi2 = 26.756 *p* < 0.001 *
		1	4	16.7%	7	53.8%	1	3.7%	
		2	9	37.5%	4	30.8%	9	33.3%	
		3	9	37.5%	-	-	17	63.0%	
		4	1	4.2%	-	-	-	-	
Intensity of positivity	DEC1	0	5	20.8%	6	46.2%	2	7.4%	Chi2 = 10.238 *p* = 0.115
		1	9	37.5%	3	23.1%	16	59.3%	
		2	9	37.5%	4	30.8%	8	29.6%	
		3	1	4.2%	-	-	1	3.7%	
	CD44	0	1	4.2%	2	15.4%	-	-	Chi2 = 11.658 *p* = 0.070
		1	6	25.0%	5	38.5%	2	7.4%	
		2	8	33.3%	3	23.1%	12	44.4%	
		3	9	37.5%	3	23.1%	13	48.1%	
Immunoscore	DEC1 (mean ± SD; min ÷ max; mediana)		3.21 ± 2.919 0 ÷ 9 2.50		2.00 ± 2.415 0 ÷ 8 3.00		3.48 ± 2.592 0 ÷ 12 3.00		Kruskal–Wallis H = 3.278 *p* = 0.194
	CD44 (m ± SD; min ÷ max; mediana)		4.87 ± 2.894 0 ÷ 12 6.00		2.38 ± 2.293 0 ÷ 6 1.00		6.22 ± 2.154 2 ÷ 9 6.00		Kruskal–Wallis H = 16.127 *p* < 0.001 *

* Significant differences (*p* < 0.05); SD—standard deviation.

**Table 6:** Intergroup comparison of extent of positivity, intensity of positivity, and immunoreactive scores.

			Types of Dysplasia								Pearson Chi-Squared Test
			0		1		2		3		
			N	%	N	%	N	%	N	%	
Extent of positivity	DEC1	0	6	31.6%	2	10.5%	3	15.8%	2	28.6%	Chi2 = 14.083 *p* = 0.295
		1	2	10.5%	4	21.1%	2	10.5%	-	-	
		2	5	26.3%	3	15.8%	6	31.6%	3	42.9%	
		3	3	15.8%	10	52.6%	6	31.6%	2	28.6%	
		4	3	15.8%	-	-	2	10.5%	-	-	
	CD44	0	2	10.5%	1	5.3%	-	-	-	-	Chi2 = 13.340 *p* = 0.345
		1	7	36.8%	3	15.8%	2	10.5%	-	-	
		2	6	31.6%	6	31.6%	7	36.8%	3	42.9%	
		3	4	21.1%	9	47.4%	9	47.4%	4	57.1%	
		4	-	-	-	-	1	5.3%	-	-	
Intensity of positivity	DEC1	0	6	31.6%	2	10.5%	3	15.8%	2	28.6%	Chi2 = 8.715 *p* = 0.464
		1	6	31.6%	8	42.1%	9	47.4%	5	71.4%	
		2	7	36.8%	8	42.1%	6	31.6%	-	-	
		3	-	-	1	5.3%	1	5.3%	-	-	
	CD44	0	2	10.5%	1	5.3%	-	-	-	-	Chi2 = 12.083 *p* = 0.209
		1	6	31.6%	3	15.8%	4	21.1%	-	-	
		2	6	31.6%	4	21.1%	8	42.1%	5	71.4%	
		3	5	26.3%	11	57.9%	7	36.8%	2	28.6%	
Immunoscore	DEC1 (mean ± SD; min ÷ max; mediana)		2.74 ± 2.766 0 ÷ 8 2.00		3.63 ± 2.671 0 ÷ 9 3.00		3.37 ± 3.004 0 ÷ 12 3.00		1.71 ± 1.254 0 ÷ 3 2.00		Kruskal–Wallis H = 3.100 *p* = 0.376
	CD44 (m ± SD; min ÷ max; mediana)		3.42 ± 2.673 0 ÷ 9 3.00		5.47 ± 2.894 0 ÷ 9 6.00		5.58 ± 2.854 1 ÷ 12 6.00		5.86 ± 1.676 4 ÷ 9 6.00		Kruskal–Wallis H = 7.655 *p* = 0.054

SD—standard deviation.

**Table 7:** DEC1 and CD44 immunoscores in NM and OPMD.

Groups	DEC1 Immunoscores				CD44 Immunoscores			
	Mean ± SD	Min ÷ Max	Median	Mann–Whitney U Test	Mean ± SD	Min ÷ Max	Median	Mann–Whitney U Test
Group 0	1.32 ± 1.73	0 ÷ 8	1.00	*p* < 0.001 *	2.21 ± 1.94	0 ÷ 9	2.00	*p* < 0.001 *
Groups 1 + 2 + 3	3.08 ± 2.70	0 ÷ 12	3.00		4.94 ± 2.83	0 ÷ 12	6.00	

* Significant differences (*p* < 0.05); SD—standard deviation.

**Table 8:** DEC1 and CD44 immunoscores in NM and OSCC.

Groups	DEC1 Immunoscores				CD44 Immunoscores			
	Mean ± SD	Min ÷ Max	Median	Mann–Whitney U Test	Mean ± SD	Min ÷ Max	Median	Mann–Whitney U Test
Group 0	1.32 ± 1.73	0 ÷ 8	1.00	*p* < 0.001 *	2.21 ± 1.94	0 ÷ 9	2.00	*p* < 0.001 *
Group 4	3.83 ± 2.99	0 ÷ 12	3.50		7.06 ± 3.24	0 ÷ 12	8.00	

* Significant differences (*p* < 0.05); SD—standard deviation.

## Data Availability

The data that support the findings of this study are available upon request from the corresponding author.
